# Biological distinction between grades 2 and 3 with respect to intravesical recurrence in T1 high-grade bladder tumors: a retrospective study

**DOI:** 10.1186/s12894-022-01000-z

**Published:** 2022-04-12

**Authors:** Akinaru Yamamoto, Atsunari Kawashima, Toshihiro Uemura, Gaku Yamamichi, Eisuke Tomiyama, Yoko Koh, Makoto Matsushita, Taigo Kato, Koji Hatano, Motohide Uemura, Norio Nonomura

**Affiliations:** Department of Urology, Osaka University Graduate School of Medicine, Yamadaoka 2-2, Suita, Osaka 565-0871 Japan

**Keywords:** Non-muscle-invasive bladder cancer, Grade, WHO 1973 classification system, WHO 2004/2016 classification system, Intravesical instillation therapy, Intravesical recurrence

## Abstract

**Background:**

The pathological grading system for non-muscle-invasive bladder cancer is based on the WHO 2004/2016 classification system (low-grade: LG/high-grade: HG) and the WHO 1973 classification system (Grade 1: G1/Grade 2: G2/Grade 3: G3). Recently, the usefulness of combining both systems and classifying the tumors as LG/G1, LG/G2, HG/G2, and HG/G3 has been demonstrated. In this study, we compared the prognosis of intravesical recurrence in relation to different treatment intensities between HG/G2 and HG/G3 bladder cancers.

**Methods:**

We retrospectively evaluated the clinical and therapeutic outcomes of 145 patients diagnosed with T1 HG bladder cancer between 2000 and 2020. We classified 145 patients into three groups: (1) patients with T1 HG/G2 and HG/G3 who received intravesical instillation therapy (n = 76), (2) patients with T1 HG/G2 who did not receive intravesical instillation therapy (n = 32), and (3) patients with T1 HG/G3 who did not receive intravesical instillation therapy (n = 37).

**Results:**

The median intravesical recurrence-free survival for all patients was 34.2 months. The number of tumors, the presence of intravesical instillation therapy, and tumor grade were significant prognostic factors for intravesical recurrence in all cases. Groups 2 and 3 showed significantly worse prognosis than group 1 in the multivariate analysis.

**Conclusions:**

Regarding intravesical recurrence, intravesical instillation therapy is necessary for both T1 HG/G3 and T1 HG/G2 bladder cancers.

**Supplementary Information:**

The online version contains supplementary material available at 10.1186/s12894-022-01000-z.

## Background

According to GLOBOCAN, bladder cancer is among the most prevalent cancers worldwide, ranking sixth among men with 440,864 cases and 14th among women with 132,414 cases in 2020 [1]. Bladder cancer is categorized as non-muscle-invasive bladder cancer (NMIBC) and muscle-invasive bladder cancer (MIBC) based on the results of clinical T staging [2], and both groups have different clinical outcomes and therapeutic options. NMIBC is generally associated with a 5‐year survival rate of > 88% [3]. However, it is characterized by a high recurrence rate. The probability of intravesical recurrence in cases of NMIBC ranges from 15 to 61% after 1 year and 31–78% after 5 years, and 1–45% of NMIBCs will progress to MIBC after 5 years [4]. As MIBCs require radical treatment (cystectomy, radiotherapy), prediction of recurrence and progression from NMIBC to MIBC remains an important topic of research [5].

The European Organization for Research and Treatment of Cancer (EORTC) developed a risk-stratification tool to predict the likelihood of recurrence and progression at 1 and 5 years after transurethral resection of bladder tumors (TURBT) for NMIBC in 2006 [3]. These risk groups are based on sex, age, tumor size and extent (defined as T in TNM staging), concomitant carcinoma in situ (CIS), tumor grade based on the WHO 1973 classification system (G1/G2/G3), number of tumors, and recurrence status [3]. In the most recent European Association of Urology (EAU) guidelines for NMIBC, the pathological grading of NMIBC was based on the WHO 2004/2016 classification system (LG/HG) and the WHO 1973 classification system (G1/G2/G3), which was reconstructed in 2021 based on reports suggesting that a combination of both classification systems (LG/G1, LG/G2, HG/G2, HG/G3) were more effective in predicting prognosis [6–8]. However, to the best of our knowledge, no study on bladder cancer has examined the validity of the HG/G2 and HG/G3 grades in terms of the risk of intravesical recurrence associated with different treatment intensities. Therefore, in this study, we aimed to evaluate the prognosis of intravesical recurrence of T1 HG/G2 and HG/G3 bladder cancers and to examine the biological differences in terms of intravesical recurrence between the two groups of HG bladder cancer.

## Methods

### Patients

**Fig. 1:**
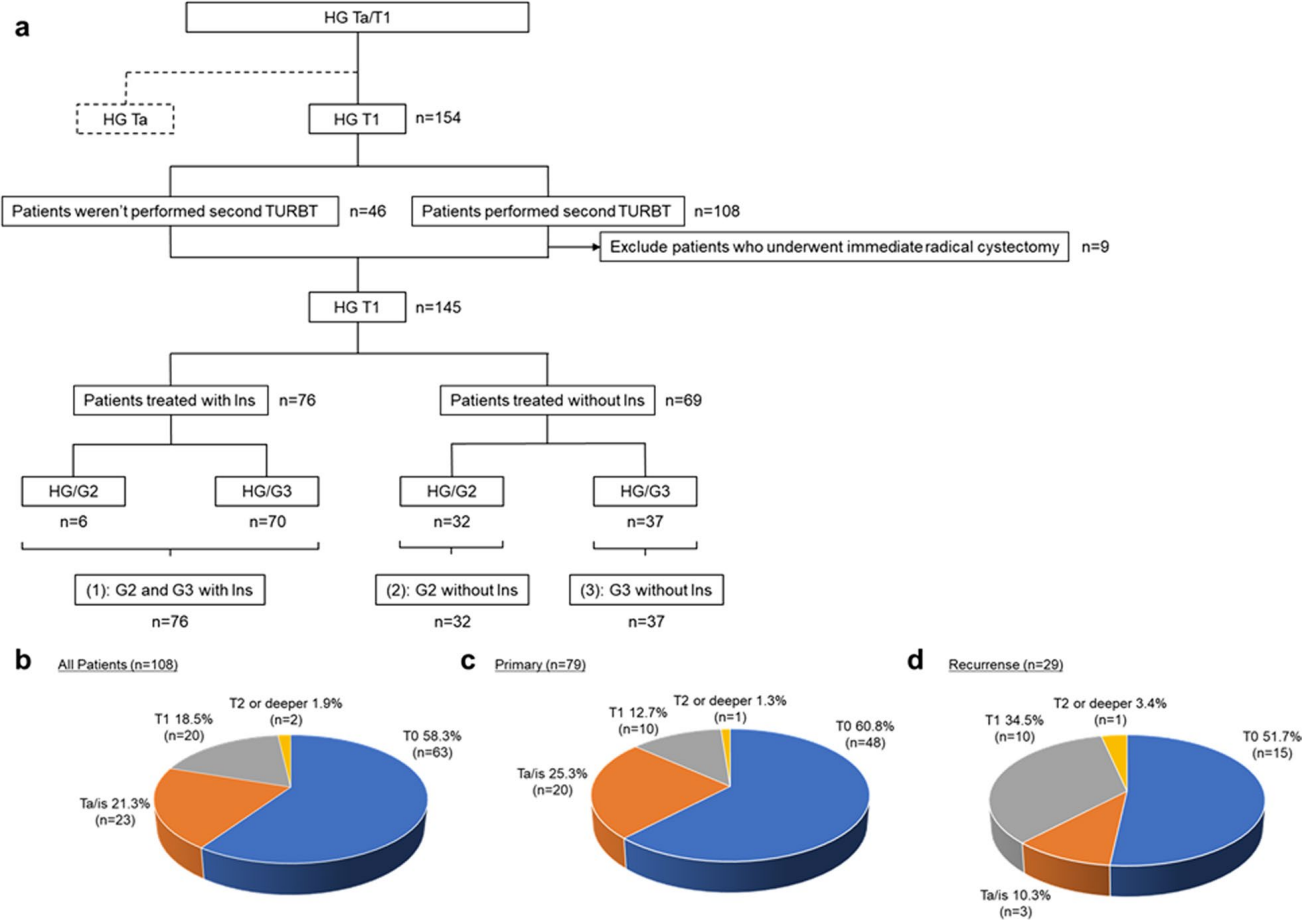
The study design and the results of transurethral resection of bladder tumors (TURBT) at our institution. **a** The protocol for the patients in this study; **b** Results of the second TURBT for all patients showed that approximately 80% of T1 patients underwent sufficiently deep resection in the initial TURBT; **c** Among patients with primary disease, more than 86% underwent sufficiently deep resection in the initial TURBT; **d** Among patients with recurrent disease, approximately 62% underwent sufficiently deep resection in the initial TURBT; *HG* high grade, *TURBT* transurethral resection of bladder tumor, *Ins* intravesical instillation therapy, *G2* grade 2, *G3* grade 3

In this study, 145 patients who underwent TURBT and were histopathologically diagnosed with T1 HG bladder cancer at Osaka University Hospital between January 2000 and July 2020 were included. First, we included 154 T1 HG cases to determine the intensity of treatment. For the evaluation of intravesical recurrence, nine patients who underwent immediate radical cystectomy were excluded from the assessment of intravesical instillation therapy (Fig. 1a). Bladder tissues were fixed in 10% buffered formalin immediately and 24 h later, embedded in paraffin, stained with hematoxylin and eosin, and diagnosed and evaluated using the WHO 2004/2016 grading system (LG/HG) and the WHO 1973 grading system (G1/G2/G3) by skilled pathologists. This single-institution, retrospective study was approved by the Institutional Review Board of Osaka University Hospital (# 3397-12).

### Study design

Risk factors for intravesical recurrence were examined, namely, sex (male vs. female), age (≤ 70 vs. > 70 years), number of tumors (single vs. multiple), size of tumors (< 3 vs. ≥ 3 cm), history of recurrence (primary vs. recurrent), presence of CIS (yes or no), the WHO 1973 classification system (G2 vs. G3), a history of a second TURBT (yes or no), and a history of intravesical instillation therapy (yes or no). Regarding the history of recurrence, “primary” is defined as the first case of naive bladder tumor, and “recurrent” is defined as the case of intravesical recurrence, which does not include recurrent cases of upper tract urothelial carcinoma. In our institution, the treatment strategy was to avoid performing either the second TURBT or intravesical instillation therapy for T1 HG/G2 bladder tumors but to perform both the second TURBT and intravesical instillation therapy for T1 HG/G3 bladder tumors. As only six patients in the G2 group received intravesical instillation therapy, we planned to investigate whether the risk of intravesical recurrence was exacerbated by the lack of intravesical instillation therapy instead of investigating whether intravesical instillation therapy prolonged the time to intravesical recurrence. To evaluate whether the relationship between treatment intensity and WHO 1973 grade contributed to the risk of intravesical recurrence, we divided the cohort into three groups: a reference group of patients with T1 HG/G2 and HG/G3 who underwent intravesical instillation therapy (group 1; n = 76), patients with T1 HG/G2 who did not undergo intravesical instillation therapy (group 2; n = 32), and patients with T1 HG/G3 who did not undergo intravesical instillation therapy (group 3; n = 37) (Fig. 1a). We evaluated these three groups using other variables in multivariate analysis.

### Statistical analysis

Intravesical recurrence-free survival was calculated using the Kaplan–Meier method with the log-rank test using the date of TURBT at the time of initial T1 diagnosis as the starting date, and prognostic factors for intravesical recurrence were investigated using univariate and multivariate Cox proportional hazards models. The differences in parameters between the groups were assessed using Fisher's exact test. The tests were two-sided, and differences were considered statistically significant at *p* < 0.05. Statistical analyses were performed using JMP Pro 16 (SAS Institute Inc., Cary, NC, USA) and GraphPad Prism 5 (GraphPad Software, USA).

## Results

### Patient characteristics

Patient and tumor characteristics are summarized in Table 1. The study population included 117 men (81%) and 28 women (19%). The median patient age was 74 years (32–89 years); 52 patients (36%) had a single tumor, and 93 patients (64%) had multiple tumors; 116 patients (80%) had tumors smaller than 3 cm, and 29 (20%) had tumors larger than 3 cm; 103 patients (71%) had primary tumors, and 42 (29%) had recurrent tumors; and 111 patients (77%) did not show concomitant CIS, while 34 patients (23%) had concomitant CIS. Based on the WHO 1973 classification system, 38 patients (26%) were categorized as G2, and 107 (74%) were categorized as G3. A second TURBT was performed in 99 patients (68%). The results of the second TURBT at our institution showed that the residual rate of T1 or higher disease after initial TURBT was approximately 20% in all patients (Fig. 1b), with the rates being 14% for primary tumors (Fig. 1c) and 38% for patients showing recurrence (Fig. 1d). Intravesical instillation therapy in a series of treatments was performed in 76 cases (52%) (pirarubicin in two cases and BCG in 74 cases). Intravesical instillation therapy over the entire lifetime was performed in 85 cases (59%) (pirarubicin in two cases and BCG in 83 cases). Groups 1, 2, and 3 showed statistically significant differences in the history of recurrence (*p* = 0.0492), presence of concomitant CIS (*p* = 0.0001), the WHO 1973 classification system categorization (*p* < 0.0001), history of a second TURBT (*p* < 0.0001), and history of intravesical instillation therapy (in a series of treatments, *p* < 0.0001; over the whole lifetime, *p* < 0.0001). As far as data were available, there was one case of prostatic urethral tumor. The patient with prostatic urethral tumor underwent immediate total cystectomy and was excluded from this study.

**Table 1:** Characteristics of patients with histopathologically diagnosed T1 HG bladder cancers

Groups parameter	Total (n = 145)	With Ins		Without Ins		*p* value
		(1)		(2)	(3)	
		G2 (n = 6)	G3 (n = 70)	G2 (n = 32)	G3 (n = 37)	
Sex, n (%)						
Male	117 (81)	6 (100)	57 (81)	27 (84)	27 (73)	
Female	28 (19)	0 (0)	13 (19)	5 (16)	10 (27)	0.4105
Age						
Median (min–max) (yr)	74 (32–89)	75 (56–86)	73 (47–89)	74 (57–87)	76 (32–88)	0.5777
< 70, n (%)	54 (37)	2 (33)	27 (39)	9 (28)	16 (43)	
≧ 70, n (%)	91 (63)	4 (67)	43 (61)	23 (72)	21 (57)	0.4230
Number of tumors, n (%)						
Single	52 (36)	2 (33)	22 (31)	14 (44)	14 (38)	
Multiple	93 (64)	4 (67)	48 (69)	18 (56)	23 (62)	0.4639
Maximum diameter, n (%)						
< 3 cm	116 (80)	5 (83)	54 (77)	27 (84)	30 (81)	
≧ 3 cm	29 (20)	1 (17)	16 (23)	5 (16)	7 (19)	0.7623
History of recurrence, n (%)						
Primary	103 (71)	3 (50)	54 (77)	17 (53)	29 (78)	
Recurrent	42 (29)	3 (50)	16 (23)	15 (47)	8 (22)	0.0492
Concomitant CIS, n (%)						
No	111 (77)	5 (83)	43 (61)	31 (97)	32 (86)	
Yes	34 (23)	1 (17)	27 (39)	1 (3)	5 (14)	0.0001
WHO grade 1973, n (%)						
G2	38 (26)	6 (100)	0 (0)	32 (100)	0 (0)	
G3	107 (74)	0 (0)	70 (100)	0 (0)	37 (100)	< 0.0001
Second TURBT, n (%)						
No	46 (32)	2 (33)	10 (14)	24 (75)	10 (27)	
Yes	99 (68)	4 (67)	60 (86)	8 (25)	27 (73)	< 0.0001
Ins in a series, n (%)						
No	69 (48)	0 (0)	0 (0)	32 (100)	37 (100)	
Yes	76 (52)	6 (100)	70 (100)	0 (0)	0 (0)	< 0.0001
Ins in whole lifetime, n (%)						
No	60 (41)	0 (0)	0 (0)	29 (91)	31 (84)	
Yes	85 (59)	6 (100)	70 (100)	3 (9)	6 (16)	< 0.0001

*HG* high grade, *Ins* intravesical instillation therapy, *G2* grade 2, *G3* grade 3, *CIS* carcinoma in situ, *TURBT* transurethral resection of bladder tumor

### Both grade 2 and 3 bladder tumors showed poor prognosis of intravesical recurrence in the absence of intravesical instillation therapy

**Fig. 2:**
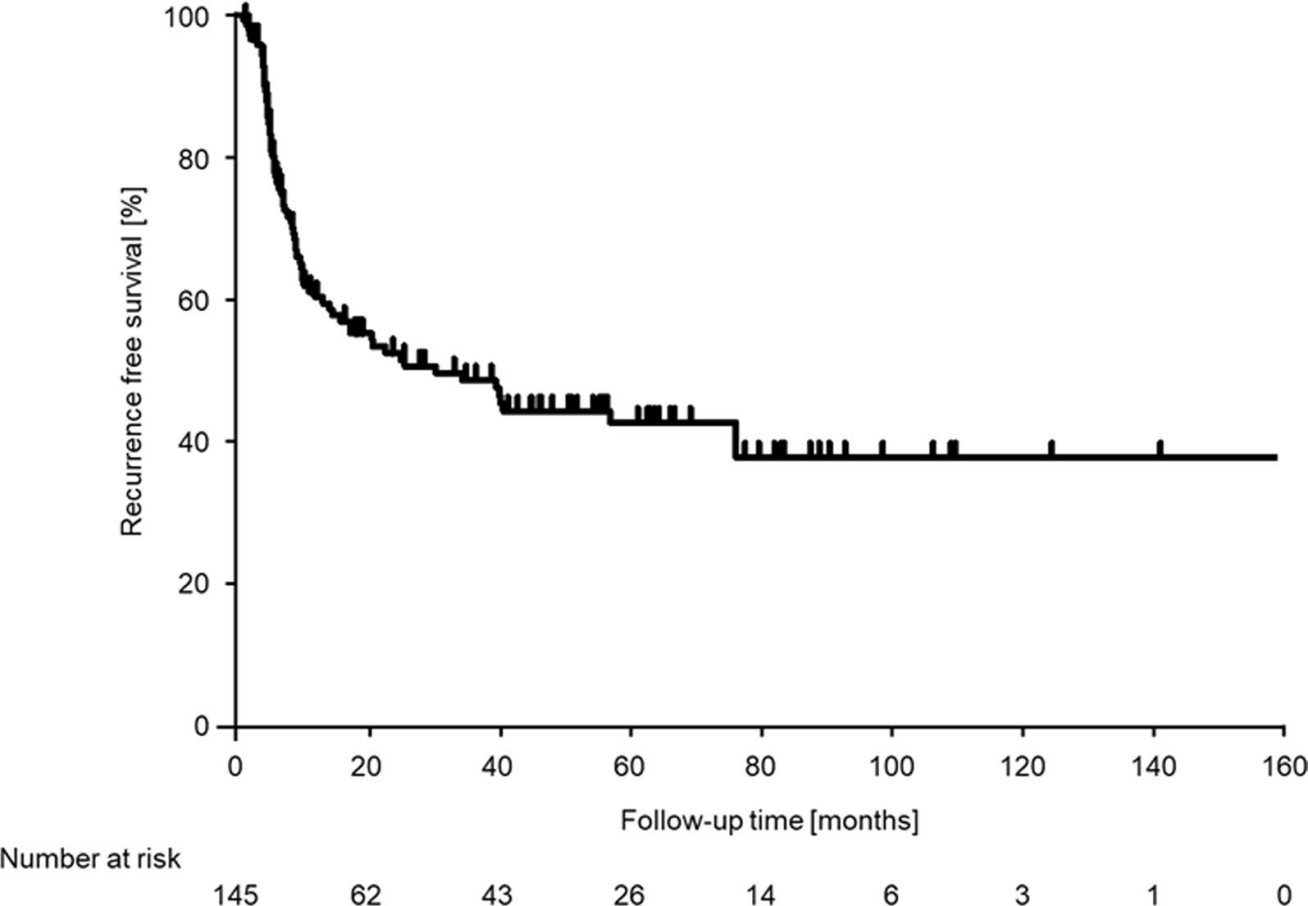
Intravesical recurrence-free survival in all patients. 5-year recurrence-free rate was 43.1%

**Fig. 3:**
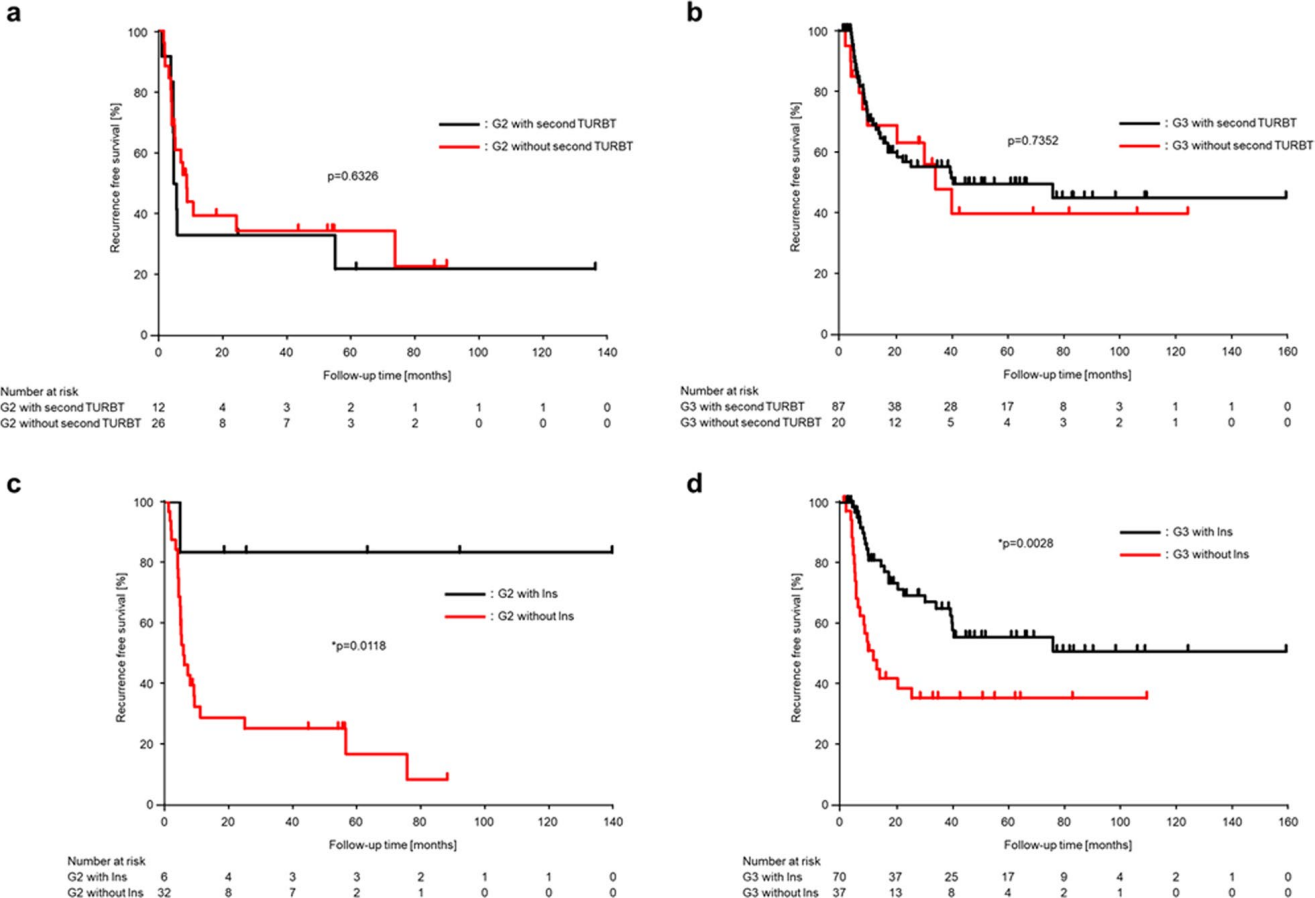
Intravesical recurrence-free survival in the G2/G3 groups with or without second transurethral resection of bladder tumors (TURBT) or Ins. **a** G2 groups showed similar intravesical recurrence-free survival with or without second TURBT; **b** G3 groups showed similar intravesical recurrence-free survival with or without second TURBT; **c** G2 groups showed significant difference with or without Ins; **d** G3 groups showed significant difference with or without Ins; *G2* grade 2, *G3* grade 3, *TURBT* transurethral resection of bladder tumor, *Ins* intravesical instillation therapy

**Fig. 4:**
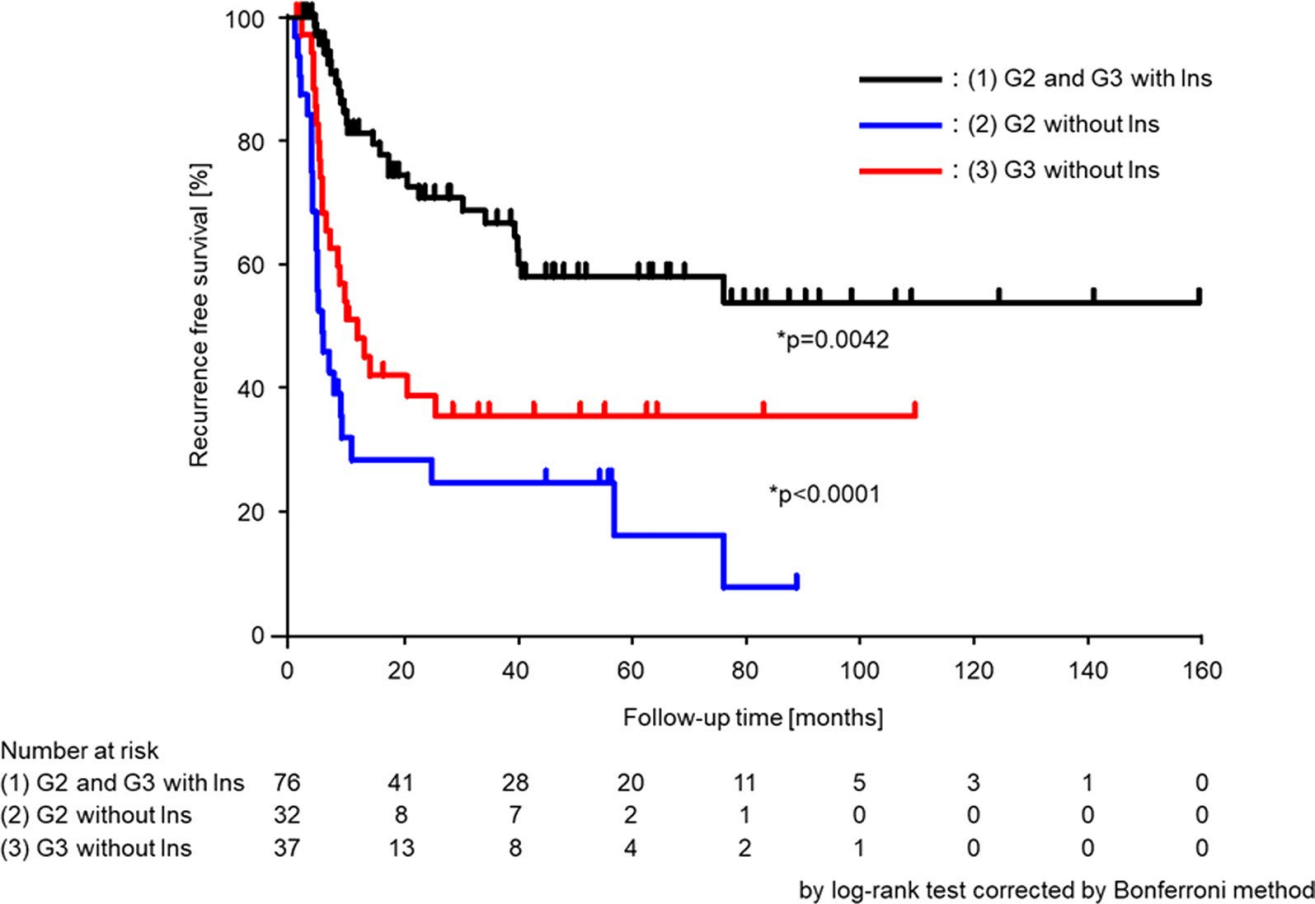
Intravesical recurrence-free survival in group 1, group 2, and group 3. The 5-year recurrence-free rate was 58.3%, 16.8%, and 35.8% for groups 1, 2, and 3, respectively, which indicated that both groups 2 and 3 had a significantly worse prognosis than group 1 (1 vs. 2, *p* < 0.0001; 1 vs. 3, *p* = 0.0042 by log-rank test corrected by Bonferroni method); *Group 1* G2 and G3 with Ins, *Group 2* G2 without Ins, *Group 3* G3 without Ins, *G2* grade 2, *G3* grade 3, *Ins* intravesical instillation

The median recurrence-free period was 34.2 months, and the 5-year recurrence-free survival rate was 43.1% for all patients (Fig. 2). Among the groups categorized by treatment intensities, in both G2 and G3 cases, the second TURBT did not contribute to intravesical recurrence (G2; *p* = 0.6326, G3; *p* = 0.7352; Fig. 3a, b), but intravesical instillation therapy significantly prolonged the time to intravesical recurrence (G2; *p* = 0.0118, G3; *p* = 0.0028; Fig. 3c, d). Univariate and multivariate analyses of the time to intravesical recurrence are presented in Table 2. The univariate analysis showed no significant differences related to sex (*p* = 0.4675) and age (*p* = 0.6423). The multivariate analysis showed no significant differences related to tumor size (*p* = 0.5982), history of recurrence (*p* = 0.3541), concomitant CIS (*p* = 0.1301), and second TURBT (*p* = 0.0771). In contrast, significant differences were observed in relation to the number of tumors (single vs. multiple: HR, 2.77; 95% CI 1.57–4.90; *p* = 0.0005) and the WHO 1973 classification system combined with intravesical instillation therapy groups (group 1 vs. 2: HR, 9.60; 95% CI 4.39–21.0; *p* < 0.0001; group 1 vs. 3: HR, 3.26; 95% CI 1.72–6.19; *p* = 0.0003) in multivariate analysis. The 5-year recurrence-free rate was 58.3% in group 1, 16.8% in group 2, and 35.8% in group 3, which indicated that both groups 2 and 3 had a significantly worse prognosis than group 1 (group 1 vs. 2: *p* < 0.0001; group 1 vs. 3: *p* = 0.0042; log-rank test corrected by Bonferroni method; Fig. 4).

**Table 2:** The risk factors of intravesical recurrence in patients with histopathologically diagnosed T1 HG bladder cancers

Variable	Univariate analysis			Multivariate analysis		
	HR	95% CI	*p* value	HR	95% CI	*p* value
Sex						
Male	1					
Female	1.23	0.70–2.14	0.4675			
Age						
< 70	1					
≧ 70	1.12	0.69–1.82	0.6423			
Number of tumors						
Single	1			1		
Multiple	1.81	1.07–3.05	0.0276	2.77	1.57–4.90	0.0005
Maximum diameter						
< 3 cm	1			1		
≧ 3 cm	1.15	0.62–2.15	0.6506	0.84	0.44–1.61	0.5982
History of recurrence						
Primary	1			1		
Recurrent	1.16	0.71–1.90	0.5576	1.29	0.75–2.21	0.3541
Concomitant CIS						
No	1			1		
Yes	0.91	0.53–1.56	0.7262	1.66	0.86–3.18	0.1301
Second TURBT						
No	1			1		
Yes	0.72	0.45–1.16	0.1728	1.72	0.94–3.15	0.0771
WHO grade 1973 and Ins						
(1) G2 and G3 with Ins	ref			ref		
(2) G2 without Ins	4.22	2.42–7.38	< 0.0001	9.60	4.39–21.0	< 0.0001
(3) G3 without Ins	2.41	1.35–4.28	0.0028	3.26	1.72–6.19	0.0003

*HG* high grade, *HR* hazard ratio, *CI* confidence interval, *CIS* carcinoma in situ, *TURBT* transurethral resection of bladder tumor, *WHO* World Health Organization, *G2* grade 2, *G3* grade 3, *Ins* intravesical instillation therapy

## Discussion

The risk of invasion and intravesical recurrence of NMIBC, which is widely recognized by the EORTC and EAU guidelines, is related to the number and size of the tumors, prior recurrence history, tumor extent (defined as T in TNM staging), the presence of concomitant CIS, and categorizations based on the WHO 1973 and 2004/2016 classification systems [3, 8–10]. In addition, the latest EAU guidelines and literature have shown that the notation method of docking the WHO 1997 classification system with the WHO 2004/2016 classification system, which yields the LG/G1, LG/G2, HG/G2, and HG/G3 classifications, is more sensitive to the prognosis of NMIBC [6–8]. In this context, LG tumors are almost always Ta [9], and the risk of recurrence was low because of the lower pathologic grade [5, 9, 11]. Thus, intravesical instillation therapy for LG bladder tumors is usually not performed, given the risks and benefits. In other words, the risk of clinically problematic invasion and intravesical recurrence should be considered mainly for HG bladder cancers, and the best we can do to reduce the risk of invasion and intravesical recurrence in incidentally encountered HG bladder tumors is to maintain appropriate treatment intensity. In our institution, we do not perform a second TURBT or intravesical instillation therapy for T1 HG/G2 bladder cancer, but we performed both treatments for T1 HG/G3 bladder cancer. We believe that predicting the prognosis associated with different treatment intensities for T1 HG bladder cancer would help determine the appropriate treatment intensity. Therefore, in this study, we limited our investigation to T1 HG bladder cancers to examine the intensity of treatment.

Among the commonly known risk factors for intravesical recurrence, the presence of only multiple tumors was a significant risk factor for intravesical recurrence in this study. This may have been due to the presence of additional treatment interventions after the initial TURBT that led to the diagnosis of T1 bladder cancer; in other words, tumors larger than 3 cm were more likely to receive a second TURBT, and intravesical instillation therapy was significantly more common in patients with concomitant CIS (Additional file 1: Fig. S1). No significant differences were observed in the history of recurrence in this study. This may be because the cohort itself was restricted to those with a high risk of recurrence and excluded Ta low-grade bladder cancers, as the comparison with recurrent cases was made with T1 HG cases only, even though they were the first cases.

In terms of treatment intensity, the second TURBT was not effective in prolonging the time to intravesical recurrence. Intravesical instillation therapy was the most important factor in prolonging the time to intravesical recurrence in this study. Several recent studies have questioned the prognostic value of a second TURBT [12, 13]. The second TURBT was originally intended to prevent the risk of underdiagnosis of tumors that were potentially MIBC [14]. The results of the second TURBT at our institution showed that the residual rate of T1 or higher disease after the initial TURBT was approximately 20% in all patients (Fig. 1b), which is consistent with most of the findings reported in the existing literature [12, 13]. In contrast, as the biological principle of intravesical recurrence is cancer seeding [15], intravesical instillation therapy to the mucosal surface of the bladder, rather than the depth of TURBT at the tumor site, can be considered to reduce the risk of intravesical recurrence or intravesical cancer seeding.

Here, we discuss the necessity of classifying T1 HG bladder cancer as HG/G2 and HG/G3. In this study, both HG/G2 and HG/G3 bladder cancers showed a significantly higher risk of intravesical recurrence if intravesical instillation therapy was not performed. Thus, in patients with T1 HG bladder cancer, intravesical instillation therapy should be performed to reduce the risk of intravesical recurrence irrespective of whether the tumor was HG/G2 or HG/G3. From this perspective, there is no need to biologically classify T1 HG bladder cancer as HG/G2 or HG/G3 when intravesical recurrence is the endpoint; these tumors can be simply qualified as T1 HG bladder cancer. Moreover, in this study, the risk of NMIBC-to-MIBC invasion or new distant metastasis (disease progression), which are more closely related to overall survival, were not significantly different between the G2 and G3 groups with and without intravesical instillation therapy (Additional file 2: Fig. S2). As for the second TURBT, it had no inhibitory effect on disease progression in the G2 and G3 groups (Additional file 3: Fig. S3). Thus, even if the endpoint is disease progression, there is little need to classify G2 and G3. The background to these conclusions is that the quality of our initial TURBT was high enough to prevent intravesical recurrence and disease progression without requiring a second TURBT (Fig. 1b). According to the latest systematic review on a second TURBT for Ta HG NMIBC, the second TURBT is useful in reducing the rates of residual disease and preventing the risk of underdiagnosis of tumors [16]. In the review, the rate of residual disease after the initial TURBT for Ta HG was 52.8% (17–67%) [16], highlighting the low rate of residual disease of the initial TURBT for T1 HG in our institution (Fig. 1c). Ruvolo compared the WHO 1973 and 2004/2016 grading systems in 35,986 Ta patients for cancer-specific mortality and concluded that the WHO 2004/2016 grading system holds a small, although measurable, advantage over the WHO 1973 grading system [17]. This indicates that the LG/HG classification is more predictive than the G1/G2/G3 classification in some populations in reducing intravesical recurrence, which is consistent with our present conclusion that HG/G2, included in HG, should be treated with intravesical instillation therapy.

This study has some limitations. First, our study was a retrospective study of patients from a single institution, and there was a large treatment bias. Second, this cohort included only T1 bladder cancer, not all NMIBC (Ta, T1), as commonly reported.

## Conclusions

In conclusion, as intravesical instillation therapy was shown to suppress intravesical recurrence in both HG/G2 and HG/G3 bladder cancer, intravesical instillation therapy can be also considered necessary for HG/G2 bladder cancer from the viewpoint of preventing intravesical recurrence.

## Supplementary Information

**Additional file 1: Fig. S1.** Difference in treatment intensity.

**Additional file 2: Fig. S2.** Disease progression free survival with or without intravesical instillation therapy before disease progression.

**Additional file 3: Fig. S3.** Disease progression free survival with or without second TURBT.

## Data Availability

The datasets used and/or analyzed during the current study are available from the corresponding author on reasonable request.

## Abbreviations

CIS: Carcinoma in situ
EORTC: The European Organization for Research and Treatment of Cancer
G1: Grade 1
G2: Grade 2
G3: Grade 3
HG: High-grade
LG: Low-grade
MIBC: Muscle-invasive bladder cancer
NMIBC: Non-muscle-invasive bladder cancer
TURBT: Transurethral resection of bladder tumors

## References

[1] World Health Organization International Agency for Research on Cancer. Bladder source: the global cancer observatory 2020. https://gco.iarc.fr/today/data/factsheets/cancers/30-Bladder-fact-sheet.pdf.

[2] Kandori S, Kojima T, Nishiyama H (2019). The updated points of TNM classification of urological cancers in the 8th edition of AJCC and UICC. Jpn J Clin Oncol.

[3] Canyilmaz E, Yoney A, Serdar L, Uslu GH, Aynaci O, Haciislamoglu E (2015). Long-term results of a concomitant boost radiotherapy technique for elderly patients with muscle-invasive bladder cancer. J Geriatr Oncol.

[4] Sylvester RJ, van der Meijden AP, Oosterlinck W, Witjes JA, Bouffioux C, Denis L (2006). Predicting recurrence and progression in individual patients with stage Ta T1 bladder cancer using EORTC risk tables: a combined analysis of 2596 patients from seven EORTC trials. Eur Urol.

[5] Babjuk M, Burger M, Compérat EM, Gontero P, Mostafid AH, Palou J (2019). European association of urology guidelines on non-muscle-invasive bladder cancer (TaT1 and carcinoma in situ)—2019 update. Eur Urol.

[6] van Rhijn BWG, Hentschel AE, Bründl J, Compérat EM, Hernández V, Čapoun O (2021). Prognostic value of the WHO1973 and WHO2004/2016 classification systems for grade in primary Ta/T1 non-muscle-invasive bladder cancer: a multicenter European Association of Urology non-muscle-invasive bladder cancer guidelines panel study. Eur Urol Oncol.

[7] Sylvester RJ, Rodríguez O, Hernández V, Turturica D, Bauerová L, Bruins HM (2021). European association of urology (EAU) prognostic factor risk groups for non-muscle-invasive bladder cancer (NMIBC) incorporating the WHO 2004/2016 and WHO 1973 classification systems for grade: an update from the EAU NMIBC guidelines panel. Eur Urol.

[8] Babjuk M, Burger M, Comperat E, Gontero P, Liedberg F, Masson-Lecomte A, et al. Non-muscle-invasive bladder cancer 2021 guideline. Available At: https://uroweb.org/guideline/non-muscle-invasive-bladder-cancer/.10.1016/j.eururo.2021.08.010 34511303

[9] Flaig TW, Spiess PE, Agarwal N, Bangs R, Boorjian SA, Buyyounouski MK (2020). Bladder cancer version 3.2020, NCCN clinical practice guidelines in oncology. J Natl Compr Cancer Netw.

[10] Jobczyk M, Stawiski K, Fendler W, Różański W (2020). Validation of EORTC, CUETO, and EAU risk stratification in prediction of recurrence, progression, and death of patients with initially non-muscle-invasive bladder cancer (NMIBC): a cohort analysis. Cancer Med.

[11] Marcq G, Hénon F, Ouzaid I, Fantoni JC, Hermieu JF, Xylinas E (2019). Active surveillance for non-muscle invasive bladder cancer. Transl Androl Urol.

[12] Calò B, Chirico M, Fortunato F, Sanguedolce F, Carvalho-Dias E, Autorino R (2019). Is repeat transurethral resection always needed in high-grade T1 bladder cancer?. Front Oncol.

[13] Matsushita Y, Watanabe K, Watanabe H, Tamura K, Motoyama D, Ito T (2020). Second transurethral resection for high-risk non-muscle invasive bladder cancer patients: a propensity score matched analysis. Jpn J Clin Oncol.

[14] Cumberbatch MGK, Foerster B, Catto JWF, Kamat AM, Kassouf W, Jubber I (2018). Repeat transurethral resection in non-muscle-invasive bladder cancer: a systematic review. Eur Urol.

[15] Miyake M, Sugano K, Sugino H, Imai K, Matsumoto E, Maeda K (2010). Fibroblast growth factor receptor 3 mutation in voided urine is a useful diagnostic marker and significant indicator of tumor recurrence in non-muscle invasive bladder cancer. Cancer Sci.

[16] Regnier S, Califano G, Elalouf V, Albisinni S, Aziz A, Di Trapani E (2022). Restaging transurethral resection in ta high-grade nonmuscle invasive bladder cancer: a systematic review. Curr Opin Urol.

[17] Ruvolo CC, Wurnschimmel C, Wenzel M, Nocera L, Califano G, Tian Z (2021). Comparison between 1973 and 2004/2016 WHO grading systems in patients with Ta urothelial carcinoma of urinary bladder. J Clin Pathol.

