# Supplementary figures and images for: Biological distinction between grades 2 and 3 with respect to intravesical recurrence in T1 high-grade bladder tumors: a retrospective study
Source: BMC Urol. 2022 Apr 12;22:59. doi: 10.1186/s12894-022-01000-z (PMC9006582; doi:10.1186/s12894-022-01000-z)

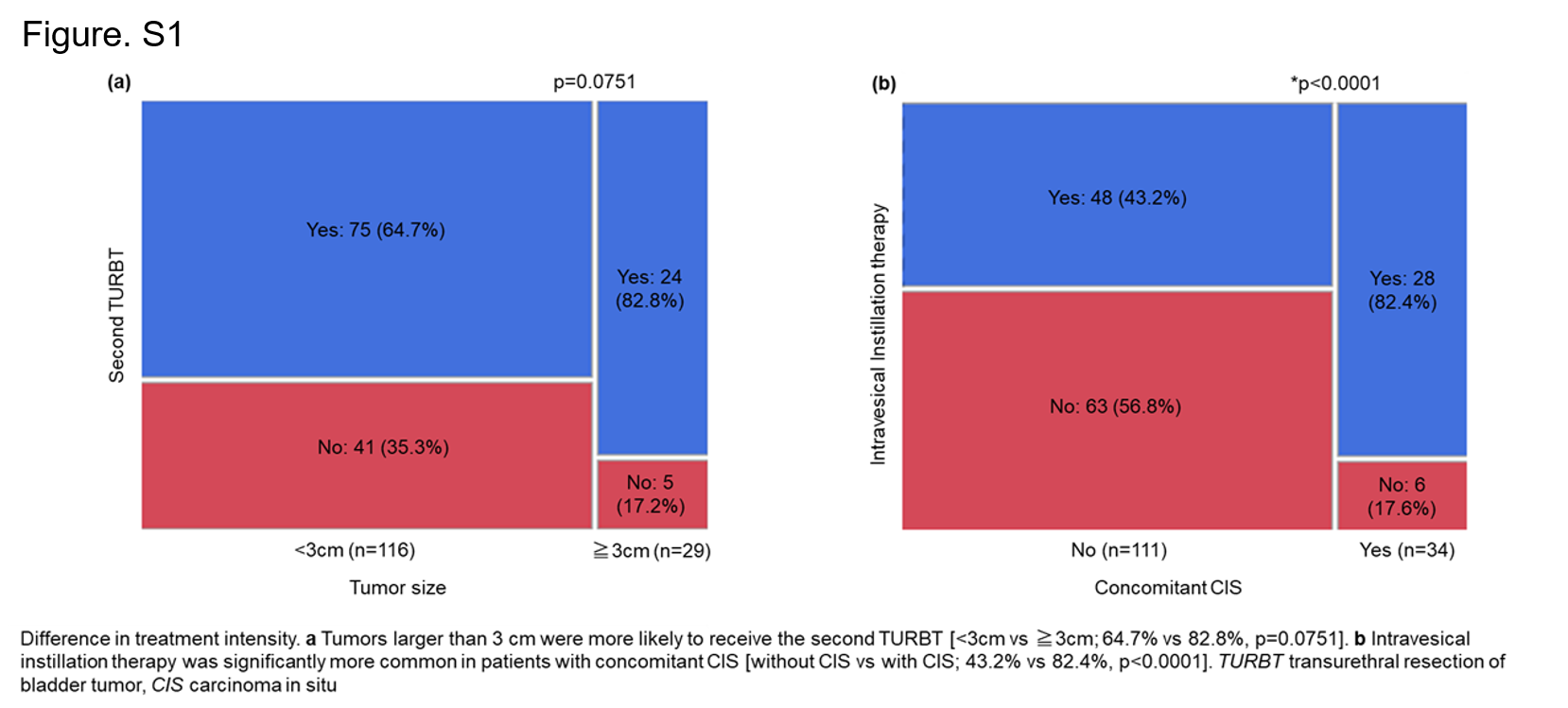

Supplement: Supplementary file 1 — Additional file 1: Fig. S1. Difference in treatment intensity. [file 12894_2022_1000_MOESM1_ESM.tif]

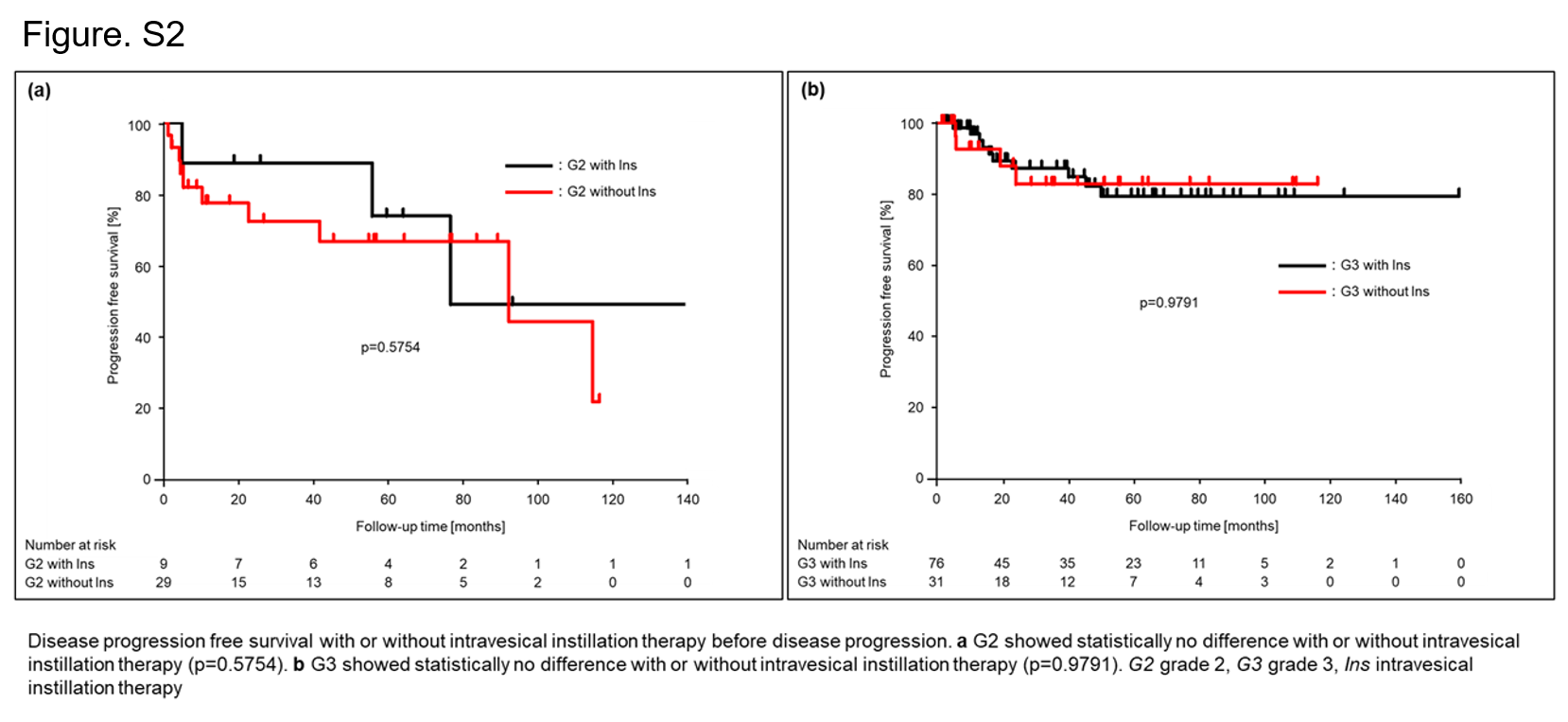

Supplement: Supplementary file 2 — Additional file 2: Fig. S2. Disease progression free survival with or without intravesical instillation therapy before disease progression. [file 12894_2022_1000_MOESM2_ESM.tif]

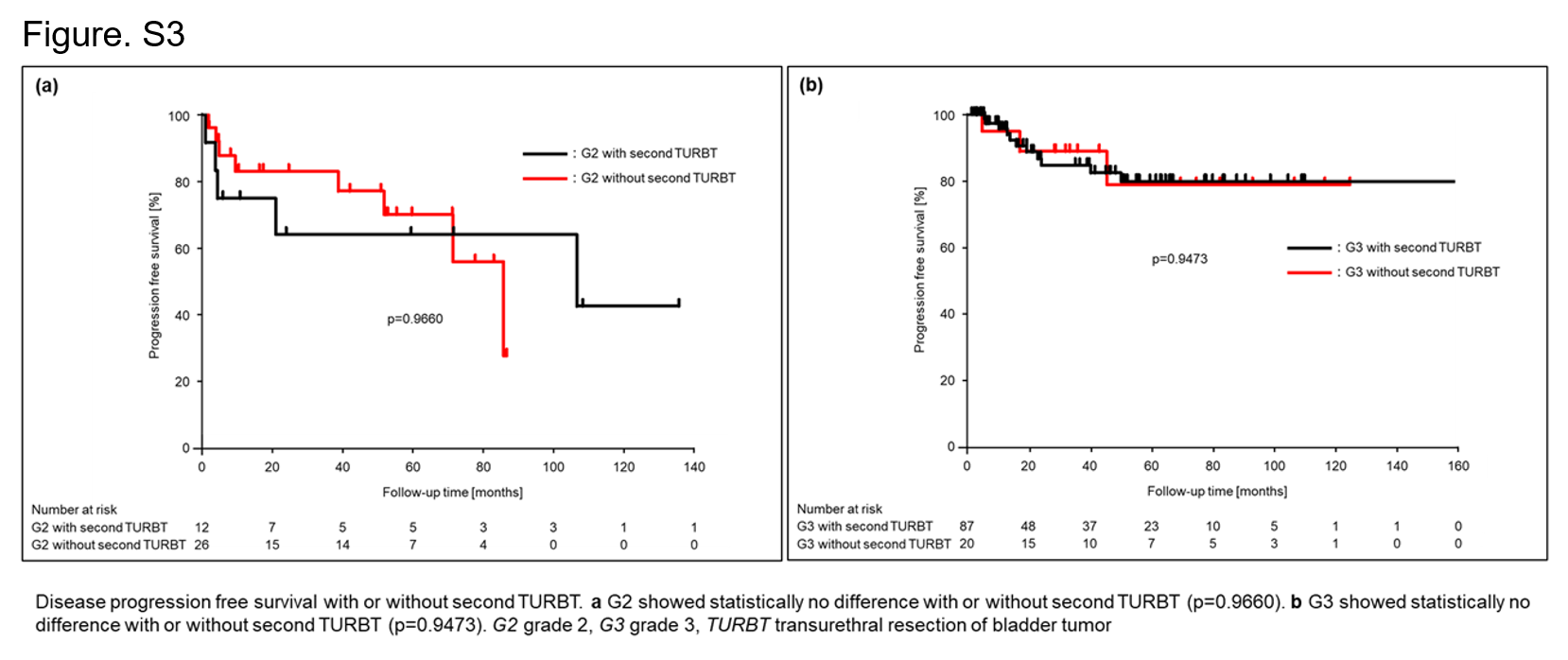

Supplement: Supplementary file 3 — Additional file 3: Fig. S3. Disease progression free survival with or without second TURBT. [file 12894_2022_1000_MOESM3_ESM.tif]
